# Can nerve regeneration on an artificial nerve conduit be enhanced by ethanol-induced cervical sympathetic ganglion block?

**DOI:** 10.1371/journal.pone.0189297

**Published:** 2017-12-08

**Authors:** Yoshiki Shionoya, Katsuhisa Sunada, Keiji Shigeno, Akira Nakada, Michitaka Honda, Tatsuo Nakamura

**Affiliations:** 1 Department of Dental Anesthesia, Nippon Dental University Hospital at Tokyo, Japan; 2 Department of Dental Anesthesiology, Nippon Dental University School of Life Dentistry at Tokyo, Tokyo, Japan; 3 Department of Bioartificial Organs, Institute for Frontier Medical Science, Kyoto University, Kyoto, Japan; University of Pennsylvania, UNITED STATES

## Abstract

This study aimed to determine whether nerve regeneration by means of an artificial nerve conduit is promoted by ethanol-induced cervical sympathetic ganglion block (CSGB) in a canine model. This study involved two experiments—in part I, the authors examined the effect of CSGB by ethanol injection on long-term blood flow to the orofacial region; part II involved evaluation of the effect of CSGB by ethanol injection on inferior alveolar nerve (IAN) repair using polyglycolic acid-collagen tubes. In part I, seven Beagles were administered left CSGB by injection of 99.5% ethanol under direct visualization by means of thoracotomy, and changes in oral mucosal blood flow in the mental region and nasal skin temperature were evaluated. The increase in blood flow on the left side lasted for 7 weeks, while the increase in average skin temperature lasted 10 weeks on the left side and 3 weeks on the right. In part II, fourteen Beagles were each implanted with a polyglycolic acid-collagen tube across a 10-mm gap in the left IAN. A week after surgery, seven of these dogs were administered CSGB by injection of ethanol. Electrophysiological findings at 3 months after surgery revealed significantly higher sensory nerve conduction velocity and recovery index (ratio of left and right IAN peak amplitudes) after nerve regeneration in the reconstruction+CSGB group than in the reconstruction-only group. Myelinated axons in the reconstruction+CSGB group were greater in diameter than those in the reconstruction-only group. Administration of CSGB with ethanol resulted in improved nerve regeneration in some IAN defects. However, CSGB has several physiological effects, one of which could possibly be the long-term increase in adjacent blood flow.

## Introduction

Functionally adequate recovery from peripheral nerve injury continues to be a clinical challenge. Recently, a bioabsorbable polyglycolic acid tube filled with collagen sponge (PGA-C) was developed [1], which has been confirmed to aid the regeneration of peripheral nerves in our experimental and clinical trials [2–6]. Blood supply to the surrounding tissue is a critical factor in nerve regeneration using PGA-C tubes [6]. In case of nerve regeneration with PGA-C tubes, success or failure is affected by blood flow in the area of reconstruction; therefore, increase in long-term blood flow to the area of nerve reconstruction through PGA-C tubes might promote nerve regeneration.

Stellate ganglion block (SGB), a type of sympathetic blockade, has several physiological effects, one of which is increased blood flow to regional tissues in the head, face, and neck, depending on its sympatholytic effects [7–9]. Previous studies have used ethanol for achieving cervical sympathetic ganglion block (CSGB) instead of SGB [10, 11]. Therefore, CSGB with ethanol might result in a long-term increase in blood flow to the region innervated by the inferior alveolar nerve (IAN). Accordingly, the objective of part I of this study was to develop a canine SGB model employing ethanol to achieve CSGB. The objective of part II was to perform electrophysiological and histological assessment of the rate of regeneration of the IAN in our canine SGB model following excision and reconstruction of the nerve using a PGA-C tube.

## Materials and methods

### Part 1: Development of the canine CSGB by ethanol

#### Surgical procedure and measurement of nasal skin temperature and oral mucosal blood flow in the mental region

These experiments employed seven male Beagles, each weighing 9.0–13.0 kg. Skin temperature was measured by infrared thermography (Neo Thermo TVS-700, NEC Avio, Tokyo, Japan; resolution, 320 × 240 pixels; thermal sensitivity, 0.08°C). Before measurement, the dogs were acclimated for 30 min in a room at 25°C. The distance between the infrared camera and the nose was 50 cm. Mean skin temperature was calculated from the thermogram using the Easy Thermo Analyzer software (Bitstrong, Tokyo, Japan). The average temperatures on the right and left sides served as baseline values.

Following thermography, the dogs were anesthetized with ketamine hydrochloride (5 mg/kg) and xylazine (1 mg/kg) and intubated. On the basis of the method of Terakawa et al. [9], mucosal blood flow was measured by laser Doppler flowmetry. The animals were placed prone, and mechanical ventilation was commenced with 1.5% sevoflurane in oxygen to achieve a stable end-tidal carbon dioxide pressure of 35 mmHg for 30 min. After hemodynamic and respiratory stabilization, left oral mucosal blood flow was measured for 1 min by laser Doppler flowmetry (ALF21, Advance, Tokyo, Japan). The flowmeter probe was fixed in position with a stent made from dental resin in order to minimize contact pressure and enable measurement at the same location each time. Blood flow was recorded continuously using the PowerLab 30 Data Acquisition System (ADInstruments, Bella Vista, Australia).

**Fig 1:**
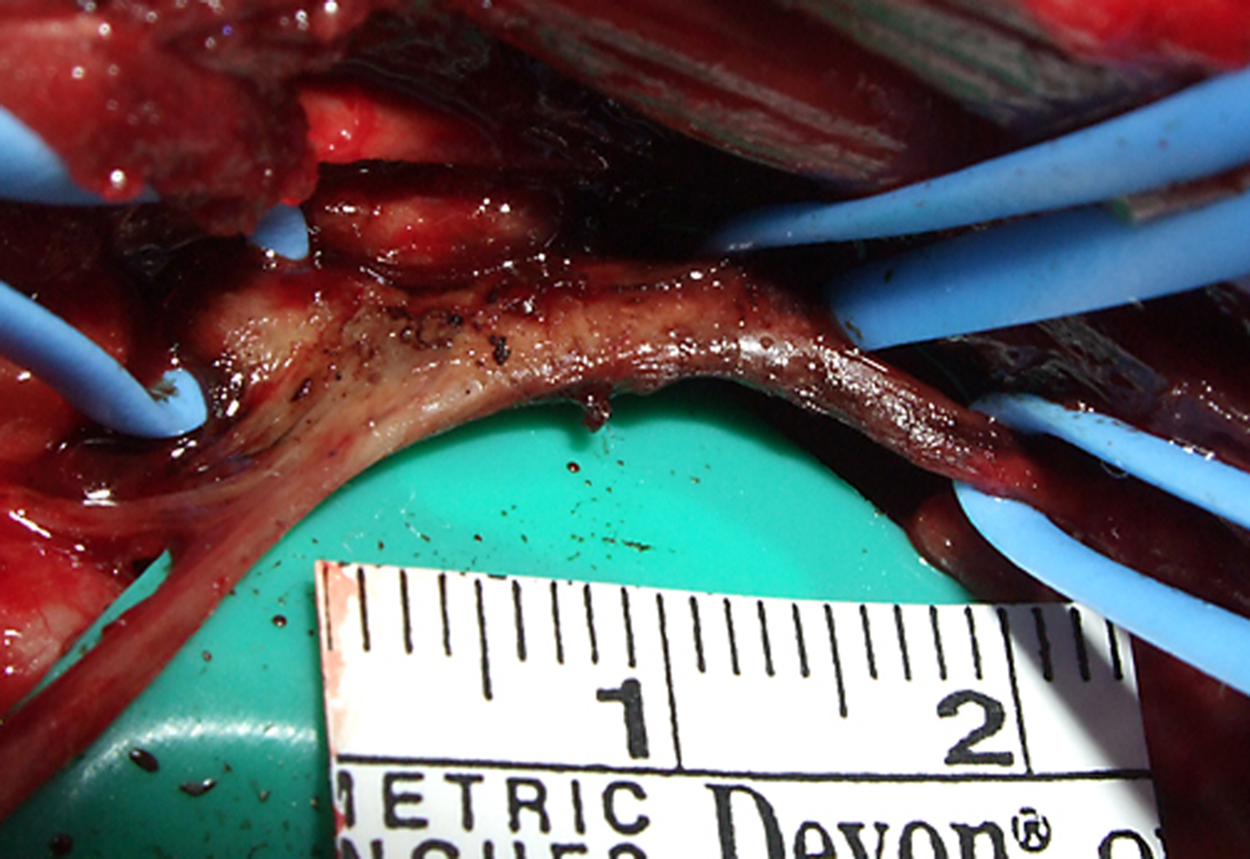
Left-side cervical sympathetic ganglion after injection of 99.5% ethanol.

Three days after baseline measurement, left CSGB was administered under ketamine hydrochloride, xylazine, and sevoflurane anesthesia. The cervical sympathetic ganglion was exposed by left lateral thoracotomy at the second intercostal space, and 0.2 mL of 99.5% ethanol was injected using a 30-gauge needle under direct visualization (Fig 1). Starting from a week after CSGB, skin temperature and oral mucosal blood flow were measured once per week for 3 months. Blood flow values were recorded as percentage change from baseline. The antibiotic isepamicin sulfate (100 mg/day) was administered for a week after surgery.

#### Histological changes in canine cervical sympathetic ganglion following ethanol block

The seven dogs were euthanized 6 months after left CSGB. The left and right cervical sympathetic ganglia were removed, fixed in 10% neutral formalin, embedded in paraffin, sectioned, and stained with hematoxylin–eosin.

### Part 2: Development and assessment of the canine model of IAN reconstruction

#### Fabrication of the nerve conduit

**Fig 2:**
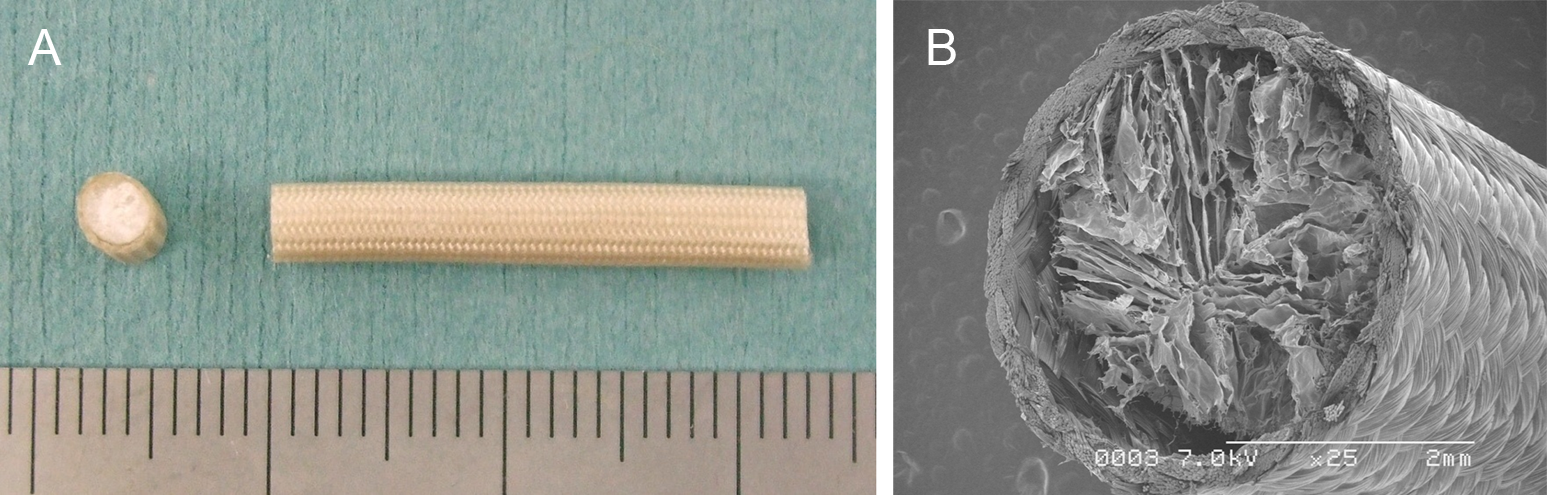
Gross image (A) and scanning electron micrograph (B) of a polyglycolic acid tube filled with collagen sponge.

An artificial nerve conduit composed of an absorbable PGA tube filled with an atelocollagen scaffold was fabricated using a tubular braiding machine equipped with 48 spindles, which carried 5 PGA fibers comprising bundles of 26 filaments. The surface of the polyglycolic acid (PGA) tube was exposed to plasma discharge to render it hydrophilic. It was then coated with amorphous collagen layers by repeated dipping into a 1% collagen hydrochloride solution, followed by drying. This coating process was repeated 10 times. The inner space of this collagen-coated tube was then filled with a collagen sponge. All collagen used in this study was atelocollagen extracted from young porcine skin by enzyme treatment (Nippon Meatpackers, Ibaraki, Japan) and tested for viruses. This particular atelocollagen mainly consists of type I collagen (70–80%), with the remainder mainly being type III collagen. The antigenicity of the atelocollagen was minimal because of the removal of antigenic telopeptides of collagen molecules during enzymatic extraction. To control the rate of bioabsorption, the PGA-C tubes were subjected to dehydrothermal treatment (140°C; 24 h) in vacuo, which resulted in the crosslinking of collagen molecules. The final length of the tube was 14 mm, with the inner diameter and wall thickness being 3–4 mm and 5.0 × 10^−5^ m, respectively (Fig 2A and 2B). Before surgery, the tube was sterilized using ethylene oxide and cut in accordance with demand in the operating room.

#### Surgical procedure for the reconstruction-only group

**Fig 3:**
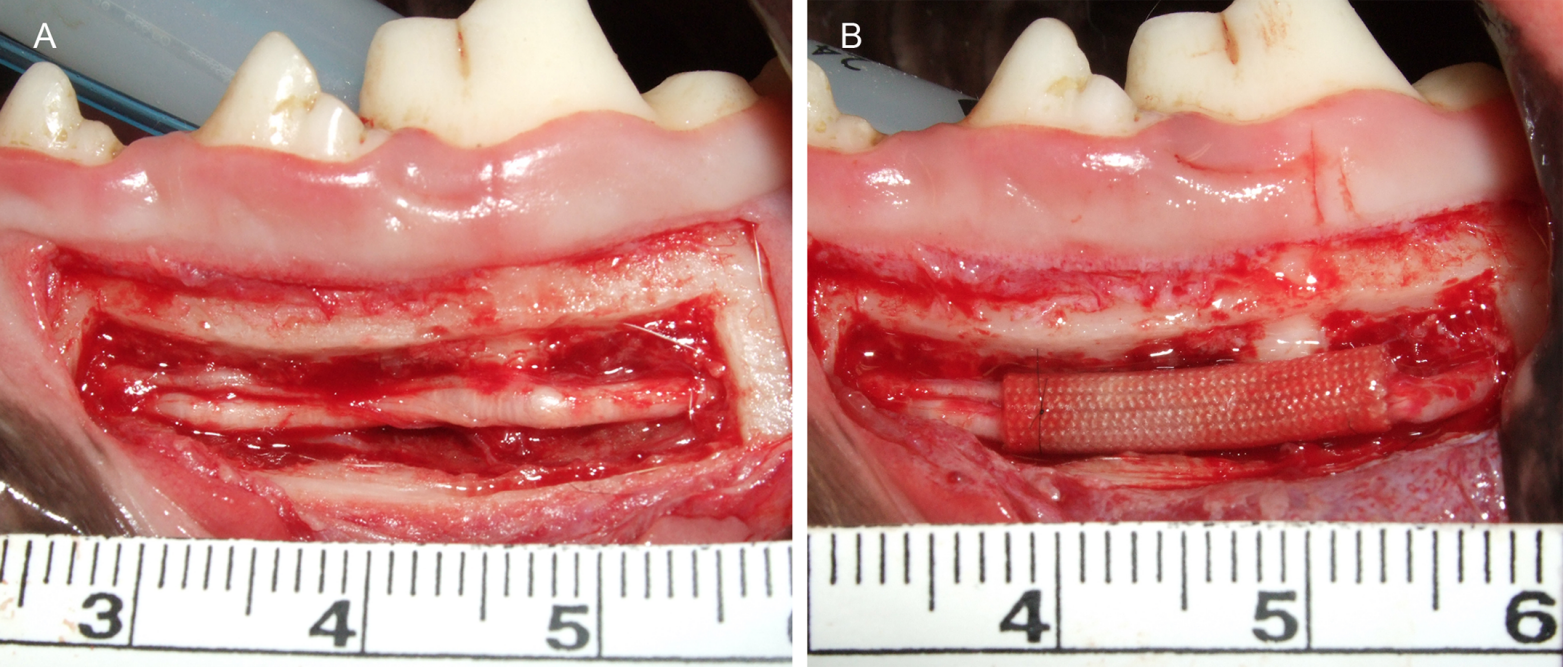
(A) Pre-reconstruction image of the left inferior alveolar nerve (IAN) exposed by removal of bone. (B) Post-reconstruction image of the left IAN reconstructed using a polyglycolic acid–collagen tube.

The mandibles of seven male Beagles were exposed by a 5-cm transverse incision in the left mandibular gingiva under general anesthesia with ketamine hydrochloride, xylazine, oxygen, and sevoflurane. The proximal aspect of the mandible was then ground into a 3-cm × 8-mm rectangle through the posterior mental foramen, using a carbide dental bur. The left IAN was exposed by carefully removing the frontal part of the mandibular bone plate (dimensions, 3-cm × 8-mm; Fig 3A). The site corresponding to the root apex of the first molar was established as the reconstruction site. The IAN was then transected with a scalpel, and a 10-mm segment was removed. The proximal and distal stumps of the severed nerve were both inserted into the nerve tube to a depth of 2 mm. The tube was then sutured to the proximal and distal nerve ends with 8–0 epineural nylon sutures with the aid of a surgical microscope (Fig 3B), and the bone plate was replaced in its original site in the mandible; the wound was then closed. The implanted PGA–collagen tube was 14 mm in length and 2 mm in diameter. The antibiotic isepamicin sulfate (100 mg/day) was administered for a week after surgery.

#### Surgical procedure for the reconstruction+CSGB group

Seven male Beagles were subjected to IAN reconstruction with CSGB. Surgery was performed using the same techniques as those employed in the reconstruction only group. A week after reconstruction, left CSGB was administered with 99.5% ethanol according to the thoracotomy technique described in Part 1.

#### Electrophysiological study

Three months after reconstruction, sensory nerve action potential (SNAP) and sensory nerve conduction velocity (SCV) of the IAN were measured on both sides using the Neuropack Σ orthodromic recorder (Nihon Kohden, Tokyo, Japan). Sensory nerve action potential was determined by the average response amplitude at 10 kHz upon 20 applications of electrical stimulation. Because the dogs had to be immobilized during measurement, ketamine hydrochloride (15 mg/kg) and xylazine (7 mg/kg) were injected intramuscularly, and a heating pad was used to maintain the body temperature at 37°C. Latency was measured between stimulation and peak amplitude, with amplitude being defined as the period from the peak of the first phase to the trough of the second phase. These results were used to calculate the SCV and recovery index (peak amplitude of the left IAN of the reconstruction-only or reconstruction+CSGB group / peak amplitude of the right IAN of the reconstruction-only group) [12, 13].

#### Histological and morphological evaluation

After measurement of SCV and recovery index, the left IAN was harvested, including 1 cm of the nerve on both sides of the reconstructed site, in both treatment groups. The right IAN of the reconstruction-only group was harvested at the site corresponding to the harvest site on the left side. Harvested nerves were immersion-fixed in 2.5% glutaraldehyde in a 0.1 M cacodylate buffer solution (pH 7.4) at 48°C for 24 h. Further, postfixation was performed with 2% osmium tetroxide solution at 48°C for 4 h and potassium ferrocyanide/0.1 M phosphate buffer solution (pH 7.4) for 2 h. The nerve specimens were then dehydrated in a graded ethanol series and embedded in epoxy resin. The specimens were then cross-sectioned (0.5–1.0 μm) and stained with toluidine blue.

Using a light microscope (BZ-9000, Keyence, Osaka, Japan), the specimens were imaged at 400x magnification at the following positions along the nerves: left IAN—the center of the regenerated segment and 2 mm distal from the stump in both treatment groups; right IAN—the center of the IAN segment corresponding to the harvest site on the left side in the reconstruction-only group. Microscopy images of regions showing myelinated nerve fibers were selected. In each of these selected images, eight to ten areas of 100- × 100-μm^2^ dimensions were established in a random and non-biased manner and evaluated for myelinated nerve fiber diameter (μm) and density (count / 100 × 100 μm^2^), nerve tissue percentage (%), and G-ratio (myelinated axon diameter / myelinated nerve fiber diameter), using a commercially available software (Dynamic cell count, Keyence, Osaka, Japan). Data evaluation was performed in a non-blinded manner.

The epoxy resin-embedded specimens prepared for immunohistochemical analysis were observed by transmission electron microscopy (TEM; Hitachi H-7000, Hitachi High Technologies, Tokyo, Japan). Cross sections (thickness, 70–90 × 10^−6^ m) were prepared using an ultramicrotome and stained with Reynold’s lead citrate and uranyl acetate.

#### Immunohistochemistry

Nerve sections at the same sites as those described above were stained with anti- neurofilament and anti-S100 antibodies. Following immersion fixation in 2.5% glutaraldehyde in a 0.1 M cacodylate buffer solution (pH 7.4) at 48°C for 24 h, the nerve specimens were embedded in paraffin according to the routine procedure. For primary antibodies, mouse anti-human neurofilament protein monoclonal antibody (N1591, Dako Japan, Kyoto, Japan) was incubated with polyclonal rabbit anti-S100 antibody (Z0311, Dako Japan, Kyoto, Japan). After washing with phosphate-buffered saline, the secondary antibody was labeled with horseradish-peroxidase and incubated for 30 min. The stained cross sections (0.5–1.0 μm) were observed under a light microscope.

### Statistical analysis

Skin temperature, myelinated nerve fiber diameter and density, G-ratio, and SCV were compared using Dunnett’s test. Oral mucosal blood flow and nerve tissue percentage were compared using Steel’s test. Recovery index was compared using unpaired t-test. The level of statistical significance was set at 5% (P < 0.05; Kyplot, KyensLab, Tokyo, Japan).

### Ethics statement

This study was conducted in accordance with the Guiding Principles in the Care and Use of Animals and approved by the Committee for Animal Research of Kyoto University (Kyoto, Japan; authorization numbers: R-16-16). All sections of this report adhere to the ARRIVE (Animal Research: Reporting of In Vivo Experiments) guidelines. Mice, rats, and rabbits have a short life expectancy and small body size. Therefore, it is not possible to perform precise surgical procedures in these animals. For this reason, we thought it necessary to use dogs. Experiments on nerve regeneration conducted using PGA-C tubes have so far used beagle dogs [1, 6]. Male beagles were purchased from SNBL, Ltd (Kagoshima, Japan) and housed at the Institute for Frontier Medical Science, Kyoto University, under controlled kennel conditions (12-h light:dark cycle). In accordance with standard care procedures, the dogs were housed in separate cages and provided solid food and water ad libitum. The veterinary staff monitored the animals on a daily basis. Intravenous fluids were used in the care of animals during all surgical procedures. Electrocardiographic readings, heart rate, and oxygen saturation were recorded during surgery. During thoracotomy, 1% lidocaine was used as a local anesthetic. Since analgesics could have influenced the experimental results, their necessity was evaluated when there were any signs of pain or distress. However, because there were no findings reminiscent of pain after surgery, analgesics were not used. In accordance with the regulations of the Committee for Animal Research, Kyoto University, a humane endpoint, such as euthanasia at an appropriate time, was to be considered for animals that experienced severe pain. At the end of the study, all dogs were euthanized by intravenous administration of an overdose of pentobarbital sodium, and all efforts were made to minimize suffering.

## Results

### Part 1: Development of the canine CSGB by ethanol

**Fig 4:**
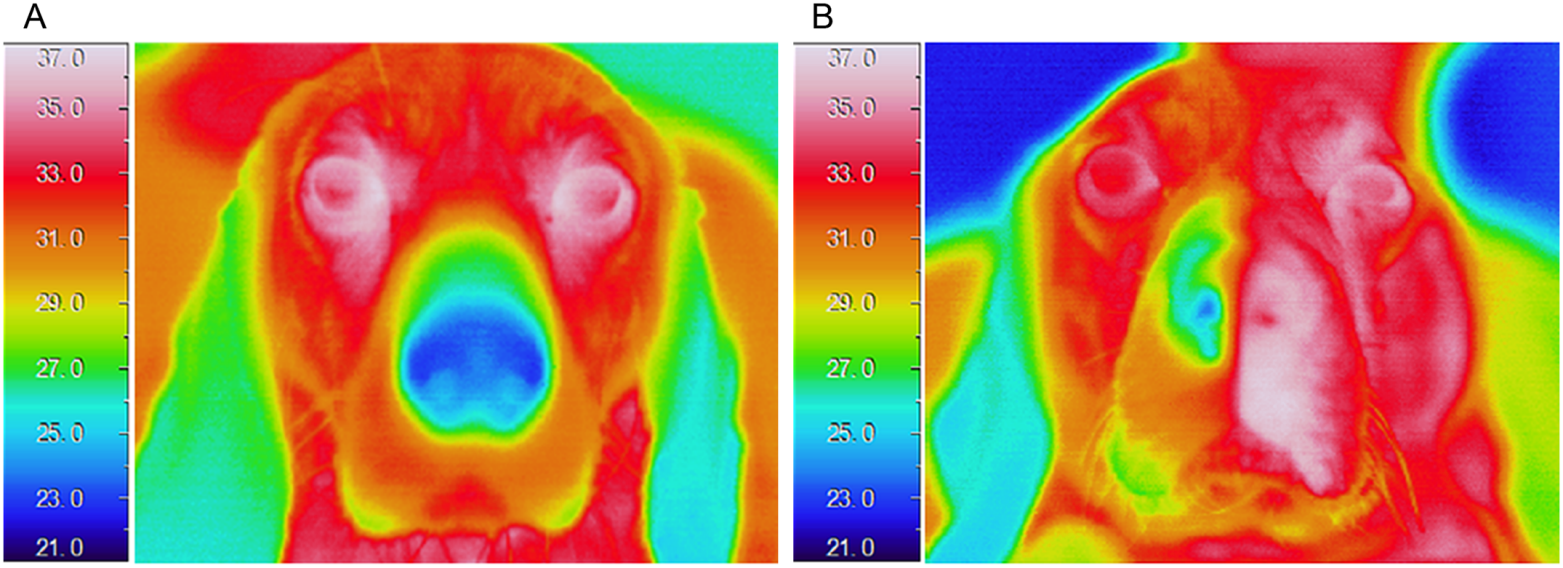
(A) Thermogram acquired before left cervical sympathetic ganglion block (CSGB) by ethanol injection. There was no difference in bilateral skin temperature. (B) A week after left CSGB, skin temperature on the left side was significantly higher compared to the baseline value.

**Fig 5:**
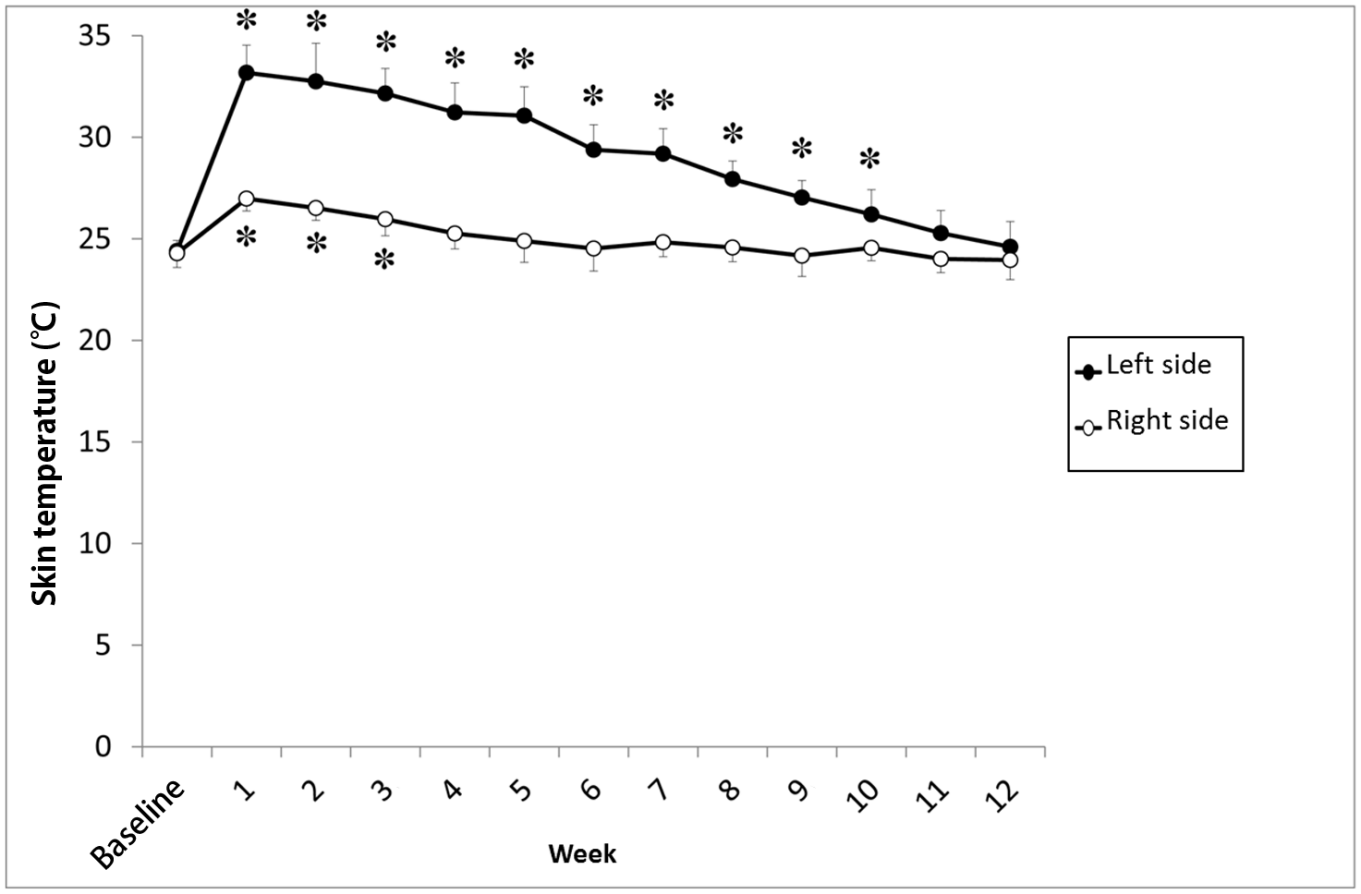
Changes in average nasal skin temperature after left cervical sympathetic ganglion block (CSGB). There was a significant increase in average nasal skin temperature after CSGB, which lasted for 10 weeks on the left side and 3 weeks on the right side. Values are expressed as mean ± SD. *P < 0.01 in comparison with the baseline value.

**Fig 6:**
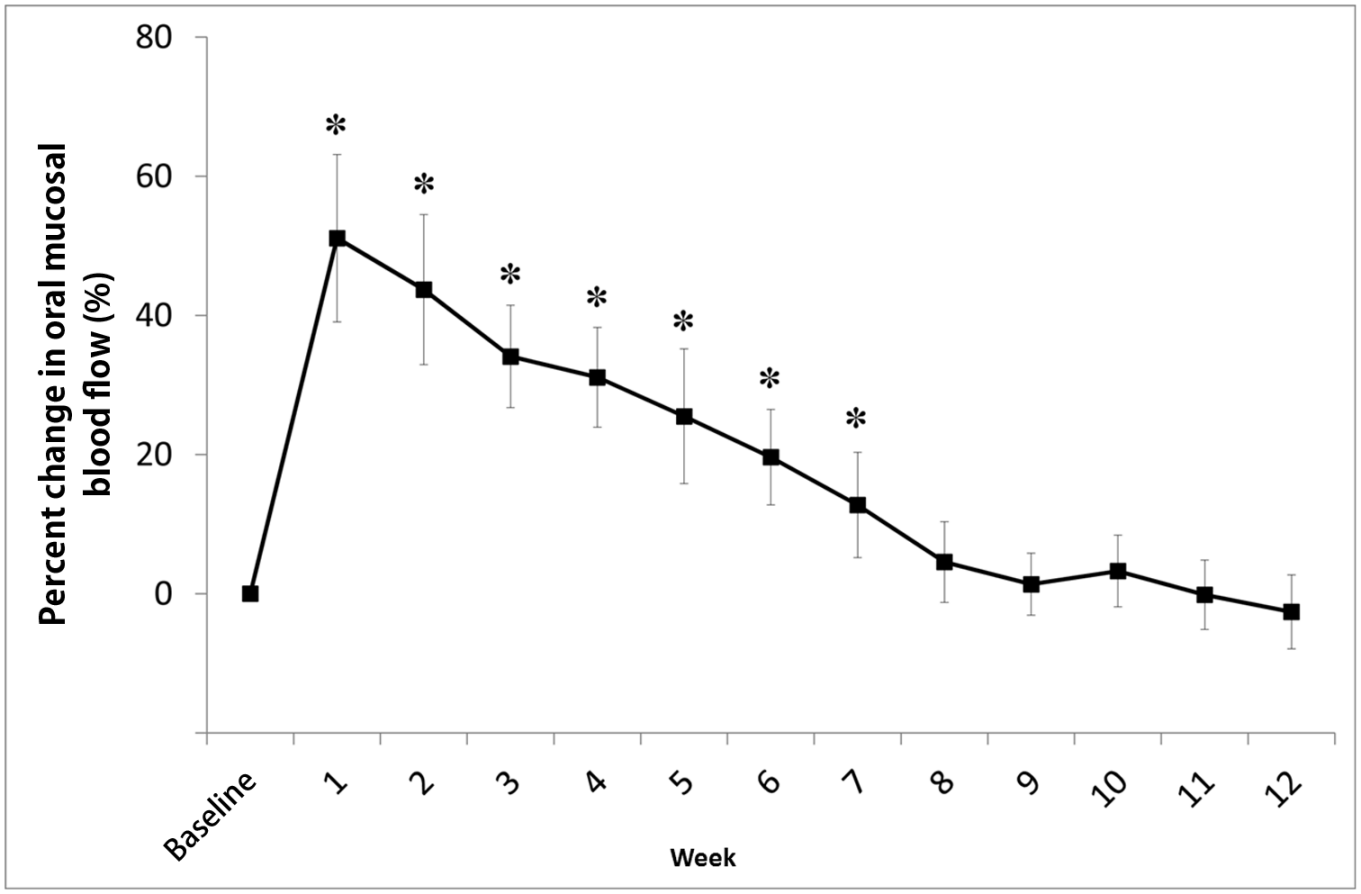
Percent changes in oral mucosal blood flow in the left mental region after cervical sympathetic ganglion block (CSGB). Blood flow was measured in the region dominated by the left mental arterial branch. The proportion of oral mucosal blood flow on the left side after CSGB remained significantly higher than that at baseline for up to 7 weeks. Values are expressed as mean ± standard deviation; *P < 0.01 in comparison with the baseline value.

A week after CSGB, nasal skin temperature and average oral mucosal blood flow on the left side had increased by 8.8 ± 1.1°C and 51 ± 12%, respectively, in comparison with the baseline values (Fig 4A and 4B). While the increase in average skin temperature lasted 10 weeks on the left side and 3 weeks on the right side (Fig 5), the increase in blood flow on the left side lasted for 7 weeks (Fig 6).

**Fig 7:**
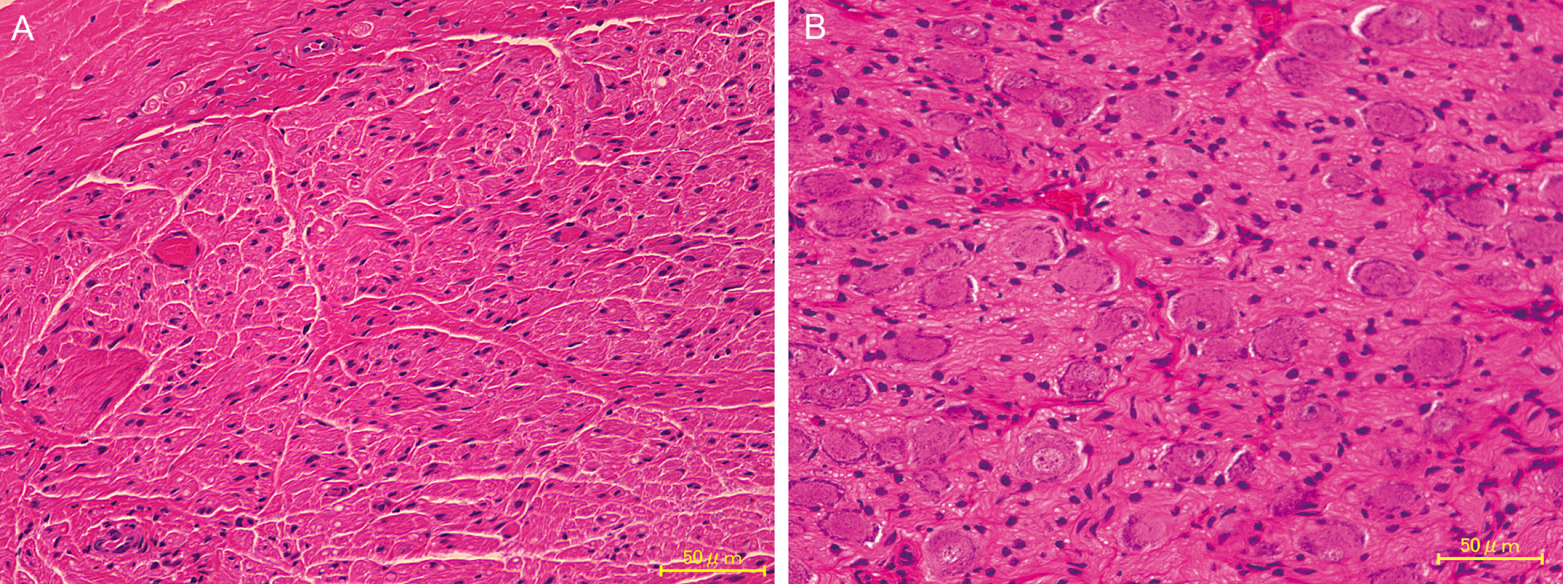
Light microscopy images of hematoxylin–eosin-stained transverse sections of the left (A) and right (B) cervical sympathetic ganglia at 6 months after ethanol injection. While the left side exhibited no ganglion cells in the entire ganglion, the right side exhibited ganglion cells throughout the ganglion. Scale bars, 50 μm.

While histological sections of the left cervical sympathetic ganglia exhibited no viable ganglion cells 6 months after ethanol injection (Fig 7A), those of the right side exhibited ganglion cells throughout the cervical sympathetic ganglia (Fig 7B).

### Part 2: Development and evaluation of the canine model of IAN reconstruction

#### Electrophysiological study

All dogs that underwent IAN reconstruction with and without CSGB were evaluated for SNAP, the results of which are summarized in Table 1. The recovery index and SCV in the reconstruction+CSGB group were significantly greater than those in the reconstruction-only group.

**Table 1:** Electrophysiological findings in the IAN at 3 months after surgery.

	Sensory nerve conduction (m/s)	Recovery index
Normal control	48.5 ± 2.8	—
Reconstruction-only group	36.8 ± 2.9*	0.22 ± 0.04
Reconstruction+CSGB group	42.0 ± 2.4*^,^^#^	0.35 ± 0.06^#^

Data are presented as mean ± standard deviation (n = 7). IAN, inferior alveolar nerve; CSBG, cervical sympathetic ganglion block.

*P < 0.05 in comparison with normal control;

^#^P < 0.05 in comparison with the reconstruction-only group

Normal control: central segment of the right IAN in the reconstruction-only group; Recovery index: peak amplitude of the left IAN of the reconstruction-only or reconstruction+CSGB group / peak amplitude of the normal control

#### Gross view and histological and morphological evaluation

**Fig 8:**
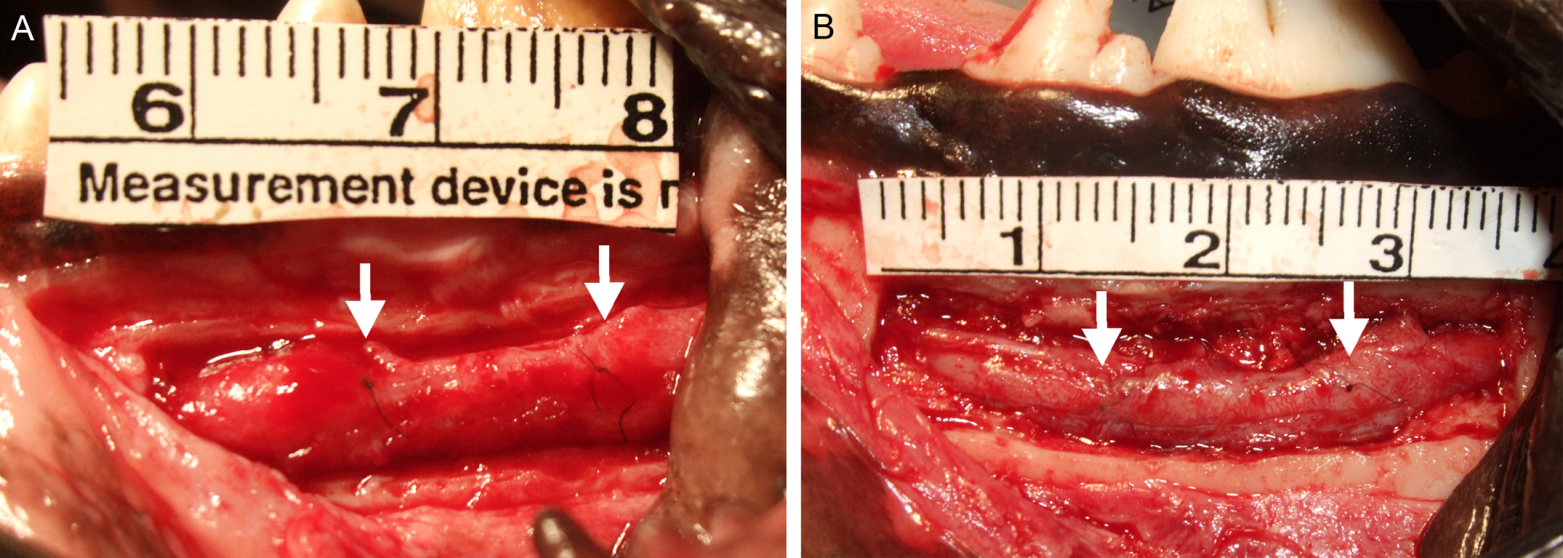
Gross images of regenerated inferior alveolar nerve in the reconstruction (A) and cervical sympathetic ganglion block (B) groups. Nerve regeneration (region between white arrowheads) was observed in both groups.

At 3 months post-reconstruction, PGA-C tubes at the reconstruction site were absorbed, and the nerve, covered by fibrous tissue, exhibited regeneration (Fig 8A and 8B). There were no differences in gross findings between the reconstruction-only and reconstruction+CSGB groups.

#### Toluidine blue staining

**Fig 9:**
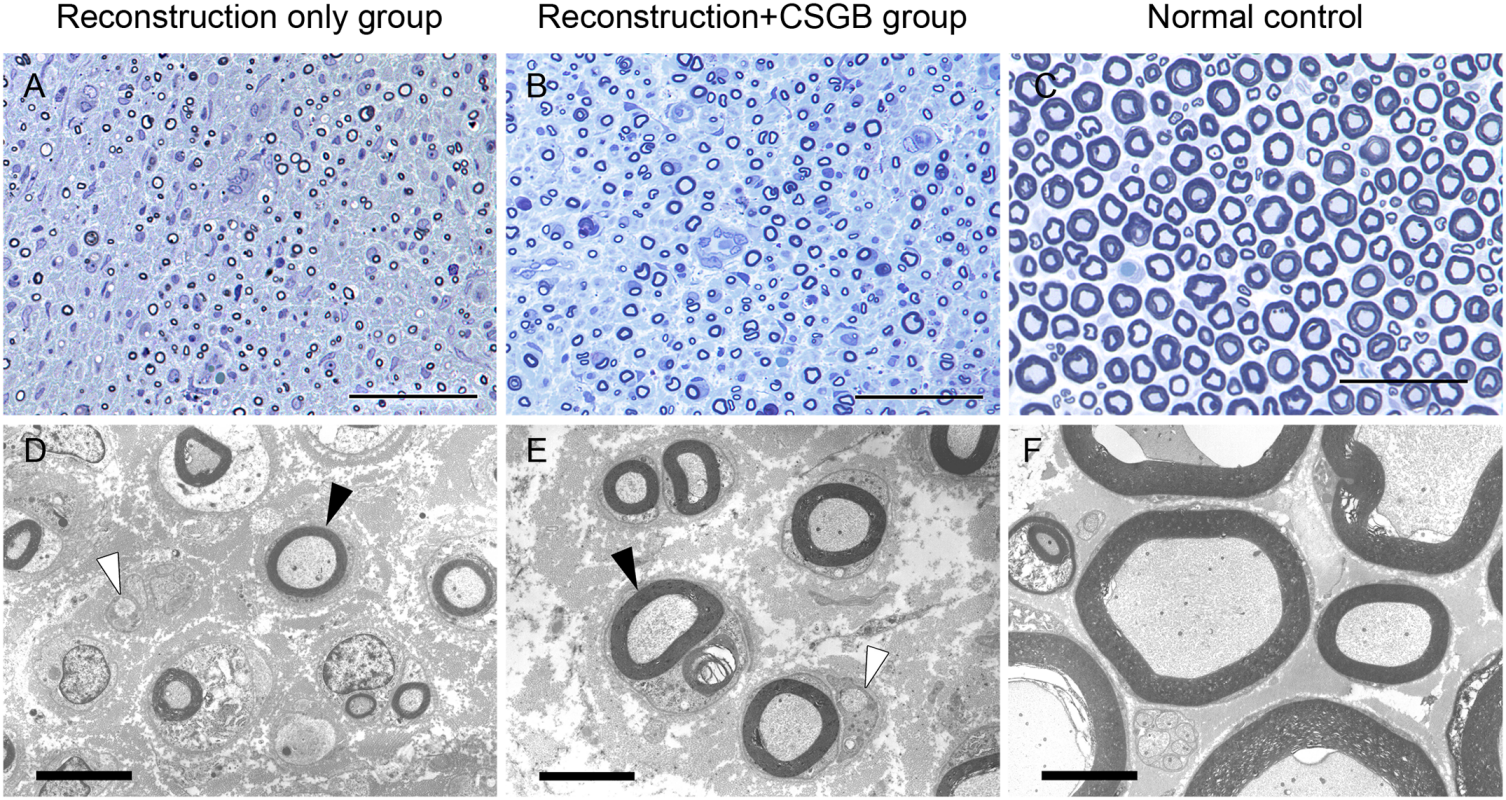
Semi-thin transverse sections (A–C; toluidine blue staining) and transmission electron microscopy images (D–F) of the regenerated inferior alveolar nerve (IAN) at 3 months post-reconstruction. Distal segments of the regenerated left IAN in the reconstruction-only (A, D) and reconstruction + cervical sympathetic ganglion block (reconstruction+CSGB; B, E) groups. (C, F) Central segment of the right IAN in the reconstruction-only group (normal control). Black and white arrowheads indicate myelinated and non-myelinated nerve fibers, respectively. Scale bars, 50 μm (A–C) and 5 μm (D–F).

Large numbers of myelinated nerve fibers were observed at the central and distal segments of the regenerated IAN in the reconstruction-only and reconstruction+CSGB groups (Fig 9A and 9B). Images of the normal control (central segment of the right IAN in the reconstruction-only group) are presented in Fig 9C.

The results of morphological evaluation are summarized in Table 2. Myelinated nerve fiber diameters and densities and nerve tissue percentages at both the central and distal segments of the regenerated left IAN in the reconstruction+CSGB group were significantly greater than those in the reconstruction-only group. The G-ratios at both the central and distal segments of the regenerated left IAN in the reconstruction+CSGB group were significantly lower than those in the reconstruction-only group.

**Table 2:** Morphological findings in the IAN at 3 months after surgery.

		Myelinated nerve fiber diameter (μm)	Myelinated nerve fiber density (count/100 μm^2^)	Proportion of nerve tissue (%)	G ratio
Normal control		8.83 ± 3.11	103 ± 8	41.3 ± 3.9	0.62 ± 0.03
Reconstruction-only group	Center	4.27 ± 1.5*	126 ± 20*	11.6 ± 2.1*	0.75 ± 0.04*
	Distal	3.47 ± 1.21*	109 ± 17*	7.3 ± 2.0*	0.74 ± 0.04*
Reconstruction+CSGB group	Center	5.11 ± 1.98*^#^	140 ± 22*^#^	15.9 ± 3.0*^#^	0.68 ± 0.05*^#^
	Distal	4.53 ± 1.36*^$^	123 ± 15*^$^	12.5 ± 2.1*^$^	0.69 ± 0.04*^$^

Data are presented as mean ± standard deviation (n = 7). IAN, inferior alveolar nerve; CSGB, cervical sympathetic ganglion block.

*P < 0.05 in comparison with normal control;

^#^P < 0.05 in comparison with the central segment of the left IAN of the reconstruction-only group;

^$^P < 0.05 in comparison with the distal end of the left IAN of the reconstruction-only group.

Normal control: central segment of the right IAN in the reconstruction-only group; G ratio: myelinated axon diameter / total myelinated fiber diameter

#### TEM

Nerve specimens of both treatment groups exhibited myelinated and unmyelinated nerve fibers and Schwann cells (Fig 9D and 9E). The reconstruction+CSGB group exhibited greater myelin sheath thickness than the reconstruction-only group. Images of the normal control are presented in Fig 8F.

#### Immunohistochemical staining

**Fig 10:**
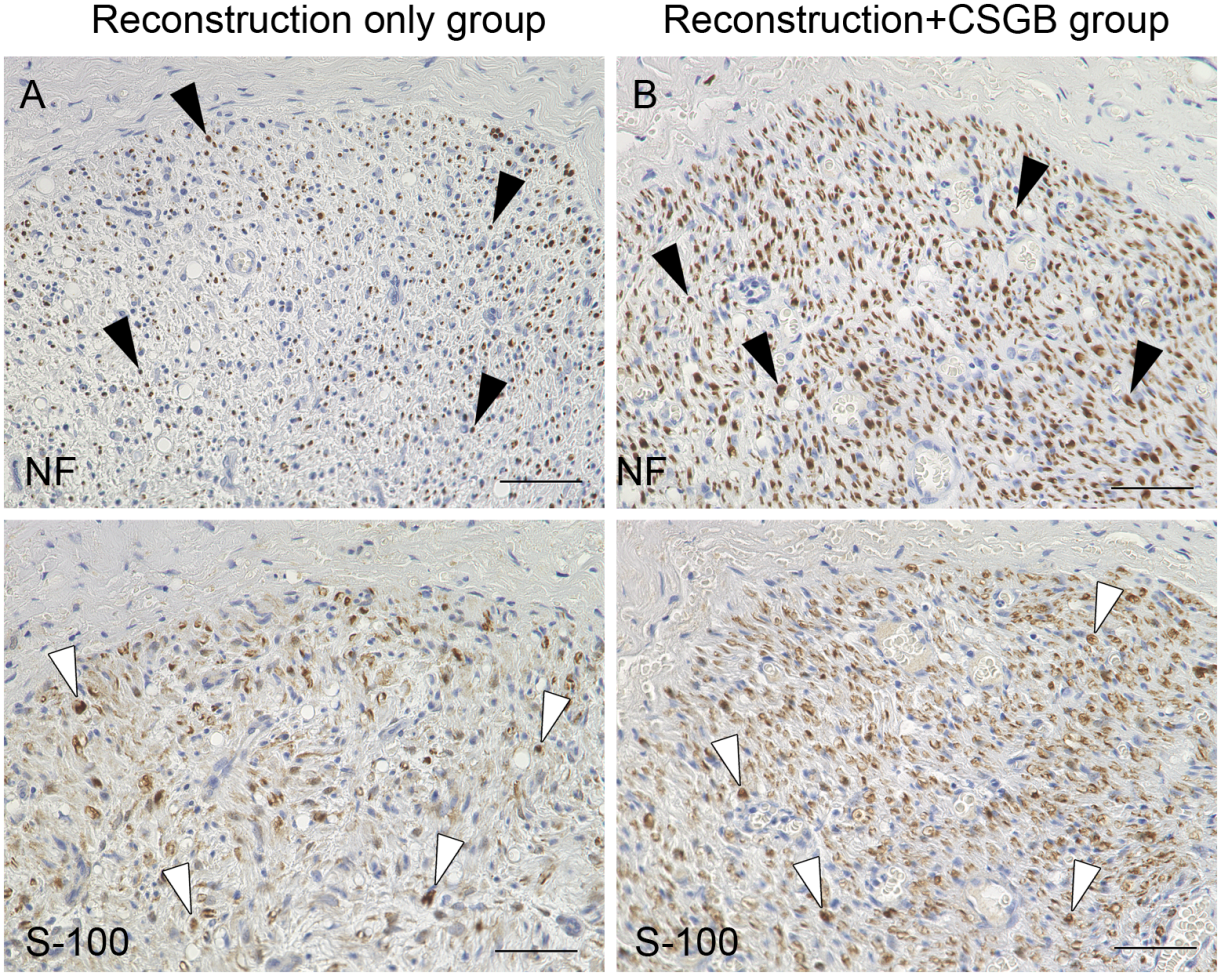
Immunohistochemistry images of distal segments of the regenerated left inferior alveolar nerve at 3 months post-reconstruction (neurofilament and S100 staining). (A, C) reconstruction-only group; (B, D) reconstruction + cervical sympathetic ganglion block (CSGB) group. Black arrowheads indicate regenerated axons. White arrowheads indicate Schwann cells. Scale bars, 50 μm.

Nerve specimens of both the reconstruction-only and reconstruction+CSGB groups showed NF-stained axons and S100-positive Schwann cells at the central and distal segments of the regenerated left IAN (Fig 10).

## Discussion

This is the first report to describe the successful promotion of nerve regeneration on an artificial nerve conduit by ethanol-induced CSGB. In comparison with the reconstruction-only group, the reconstruction+CSGB group exhibited significantly greater SCV and recovery index, which suggests that reconstruction with CSGB promoted IAN regeneration in the PGA-C tube. Myelinated nerve fiber count and diameter and myelin sheath thickness are the most reliable parameters for assessment of nerve regeneration. In addition, SNAP has been reported to be correlated with nerve fiber diameter and myelin sheath thickness [14, 15]. In regenerated nerves, the myelin sheath matures and thickens as the axon reaches the end of the distal segment [16]; thus, the relatively small G-ratio of the reconstruction+CSGB group demonstrates the progression of myelinated nerve regeneration.

When a nerve gap is so long that end-to-end tension-free suturing becomes difficult, it is necessary to employ another method for bridging the gap to allow the fibers to cross over and reach the distal nerve stump. The most widely used technique in this scenario is autologous nerve grafting. The use of autologous nerve grafts has yielded successful results in many studies [17–19], although it also has certain disadvantages such as the need for a second surgical procedure, possibility of loss of donor nerve function, limited supply of donor nerves, and dimensional mismatch between the impaired nerve and the graft [20–22]. In addition, because donor nerves are routinely obtained from a purely cutaneous source in order to minimize the resulting deficit, repaired motor axons must, by necessity, grow under less than favorable conditions [23, 24]. These disadvantages have led to the development of several alternative techniques, such as those involving the use of artificial nerve guides [25, 26].

Nakamura et al. [1] developed a PGA-collagen tube that uses a collagen scaffold as the support structure (PGA-C tube) and demonstrated that the tube allowed regeneration across a short gap of approximately 15 mm, with significantly higher levels of reinnervation than that obtained using autografts in a canine nerve-defect model. This PGA-c tube has been in clinical use since 2002 for human patients with peripheral nerve injuries and has provided some promising results [2–5].

In our study, we conducted electrophysiological, histological, and morphological evaluation at 3 months post-reconstruction. As described in our previous report [1], at 8 weeks after reconstruction of peroneal nerve deficits of 15-mm length using PGA-C tubes, most of the PGA fibers had been absorbed, and the axons ran through the structure in the proximal-to-distal direction. In a pilot study, which we conducted with reference to our previous report, when a 10-mm defect of the IAN was reconstructed with PGA-C tubes, the PGA fibers were absorbed within 3 months after surgery. Although regeneration was not yet complete at 3 months after reconstruction, it was confirmed that the axons ran through the structure in the proximal-to-distal direction. The present study focused on whether regeneration is promoted by CSGB induced by ethanol injection and not on whether regeneration is completed. Therefore, we believe that it was necessary to perform comparisons during the regeneration stage. For these reasons, we decided on the 3-month time point for electrophysiological evaluation.

Peripheral nerve regeneration requires adequate blood flow. de la Torre and Goldsmith [27] reported that transposition of the omentum increases blood flow and promotes spinal nerve fiber growth. Oxygen supply is a particularly important factor in nerve regeneration; hyperbaric oxygen treatment has been reported to promote sciatic nerve regeneration in rats [28]. The use of artificial tubes in nerve regeneration helps localize Schwann cells, which regulate the secretion of neurotrophic factors—including nerve growth factor, brain-derived neurotrophic factor, and neurotrophin-3 [29–31]—which have been reported to be beneficial for regeneration [32, 33]. In the present study, the result of S100 immunostaining revealed the presence of Schwann cells in the regenerated segment in the reconstruction+CSGB group. This result suggests that the CSGB-induced increase in blood flow aided nerve regeneration by increasing oxygen supply to the regenerated segment and, thereby, enhancing aerobic metabolism, resulting in increased ATP production and Schwann cell activity and promoting the production of neurotrophic factors. Increased blood flow has also been suggested to suppress edema at the nerve repair site and produce a favorable environment for nerve regeneration. However, CSGB has not only the effect of increasing blood flow but also other physiological effects, such as production of many types of cytokines [34, 35]. In this study, we only focused on the effect of CSGB on increasing blood flow; however, other physiological effects might have influenced the promotion of nerve regeneration. In this regard, we believe that further research is needed in the future.

In the present study, CSGB by ethanol resulted in increased blood flow in the mental arterial branch and facial artery. At 6 months after CSGB, no neurons were observed in the cervical sympathetic ganglion, which, we believe, indicates that ethanol-induced CSGB provides reliable long-term blockade of the cervical sympathetic ganglion. However, the increases in blood flow and skin temperature demonstrated by our SGB models were not permanent. Alborch et al. [36] reported that, while goats exhibited increased cerebral blood flow following removal of the superior cervical sympathetic ganglion, the proportion of blood flow returned to pre-surgical levels 15–25 days after the procedure. One conceivable reason for this phenomenon is that increase in blood flow results in vasodilation and collateral circulation, causing blood flow to shift to the contralateral side. Nasal thermography findings in the present study revealed increased skin temperature on the contralateral side as well, which indicated that increase in blood flow on the side of nerve block resulted in increased blood flow on the contralateral side through anastomosis of the left and right facial arteries.

In this study, we chose ethanol to achieve assured sympathetic ganglion block for experimental purposes. However, in clinical practice, neurolysis of the stellate ganglion poses the risk of development of permanent Horner′s syndrome (ptosis and miosis) [37]. In nerve regeneration, in order to achieve effective blood-flow increase by SGB with a local anesthetic, it is thought that several administrations of SGB are necessary. The conventionally used blind technique for SGB can cause various adverse effects, including epidural, subarachnoid, and intravascular injection, hematoma formation, and esophageal injury [38, 39]. However, in recent times, the use of ultrasound guidance has made it possible to reduce the adverse effects of SGB [40]. Noninvasive SGB administered using physical modalities such as linearly polarized near-infrared irradiation can be clinically used as an alternative to conventional invasive SGB [41, 42]. Whether noninvasive SGB using linearly polarized near infrared light is effective for increasing local blood flow requires further research; however, we believe that it holds potential as an alternative procedure.

In both treatment groups, the findings of TEM revealed several different stages of myelin sheath formation, suggesting incomplete regeneration. Moreover, the absolute values of SCV and SNAP of the regenerated IAN were lower than those of the normal control (central segment of the right IAN in the reconstruction-only group), which might have been because of the regeneration still being in progress.

The present findings could contribute to advances in surgical techniques, leading to improved outcomes of treatment for traumatic injury-induced trigeminal neuropathy using nerve guide tubes. Future studies should focus on the potential efficacy of SGB in facilitating repair at the cellular level.

This study has some limitations. We established the SGB model for an IAN injury that was completely encircled by bone. This creates a specific situation of blood flow from the three remaining sides, which is not typically seen in most cases of peripheral nerve injury. Further studies would be necessary to validate the present results in a more traditional nerve injury model. In addition, to prevent ethanol-induced damage to surrounding tissues, nerve blockade by surgical sympathectomy may be considered.

## Conclusion

Cervical sympathetic ganglion block via ethanol injection increases blood flow in the region supplied by the mental branch of the IAN. In the reconstruction+CSGB group, this increase in blood flow might have promoted nerve regeneration after reconstruction using a PGA-C tube.

## Supporting information

S1 File ARRIVE guidelines checklist. (PDF)
